# Chloroplast Responses to Drought: Integrative Mechanisms and Mitigation Strategies

**DOI:** 10.3390/ijms262411872

**Published:** 2025-12-09

**Authors:** Sanjiao Wang, Qinghua Ma, Chen Li, Sihan Zhang, Xiaomin Liu

**Affiliations:** State Key Laboratory of Tree Genetics and Breeding, College of Biological Sciences and Technology, Beijing Forestry University, Beijing 100083, China; m17835729632@163.com (S.W.); 17860631335@163.com (Q.M.); lichen0727999@outlook.com (C.L.); baijiu051006@163.com (S.Z.)

**Keywords:** molecular mechanisms, chloroplast structure and organization, antioxidant defense, functional regulators, molecular breeding

## Abstract

Drought is one of the most severe abiotic stresses limiting agricultural productivity and threatening global food security. As the central organelle responsible for photosynthesis and stress perception, the chloroplast is highly sensitive to drought, and its structural and functional stability directly determines plant adaptability. Recent studies have revealed that chloroplasts undergo pronounced ultrastructural alterations under drought stress, including thylakoid membrane shrinkage, disorganization of grana stacks, and accumulation of reactive oxygen species (ROS). Excessive ROS production causes oxidative damage to lipids, proteins, and nucleic acids, whereas moderate ROS levels act as retrograde signals to regulate nuclear gene expression. In parallel, calcium (Ca^2+^) oscillations and retrograde signaling pathways—such as those mediated by GENOMES UNCOUPLED PROTEIN1 (GUN), 3′-phosphoadenosine-5′-phosphate (PAP), and Methylerythritol cyclodiphosphate (MecPP)—integrate chloroplast-derived stress cues with nuclear responses. To counteract drought-induced damage, plants activate a series of antioxidant systems—both enzymatic (Superoxide Dismutase (SOD), Ascorbate Peroxidase (APX), Catalase (CAT)) and non-enzymatic (Ascorbic Acid (ASA), (Glutathione) GSH, tocopherols, carotenoids)—along with protective proteins such as fibrillins (FBNs) and WHIRLYs that stabilize thylakoid and membrane structures. In addition, autophagy and plastid degradation pathways selectively remove severely damaged chloroplasts to maintain cellular homeostasis. Exogenous substances, including melatonin, 5-aminolevulinic acid (ALA), and Zinc oxide (ZnO) nanoparticles, have also been shown to enhance chloroplast stability and antioxidant capacity under drought stress. In this review, we discuss the structural and functional changes in chloroplasts, signaling networks, and protective repair mechanisms under drought stress. Furthermore, we highlight future research prospects for enhancing plant stress resilience through multi-omics integration, application of functional regulators, and molecular design breeding.

## 1. Introduction

Drought is one of the most severe abiotic stresses threatening global agricultural productivity, posing persistent challenges to food security and ecosystem stability. More than 70% of a plant’s fresh biomass is composed of water, which plays an essential role in numerous physiological processes, including growth, development, and metabolic reactions. However, insufficient groundwater, infrequent rainfall, and inadequate irrigation often lead to a shortage of available water for plants. Plants frequently experience water deficit or drought stress when soil water availability declines and the rates of transpiration and evaporation are high [1]. In natural ecosystems, drought and heat stress often occur simultaneously as a closely coupled combined stress, whose detrimental effects on plants far exceed the sum of each individual stress, thus posing a substantial threat to plant survival [2,3].

Relevant studies have indicated that the photosynthetic system is particularly sensitive to the synergistic stress of drought [4]. As the primary site of photosynthesis, the chloroplast is among the most sensitive organelles within plant cells [5,6]. From the initial perception of drought signals to the occurrence of chloroplast adaptive changes, plants initiate a series of ordered and precisely regulated physiological cascade responses. On the one hand, under drought stress, plant transpiration is reduced, leading to stomatal closure and decreased CO_2_ supply. This further causes an imbalance between light reactions and dark reactions, ultimately triggering reactive oxygen species (ROS) burst [6]. On the other hand, when soil water content drops to the critical threshold, the pressure potential of root cell walls decreases, leading to insufficient cell water absorption and subsequent significant reduction in the water potential of mesophyll cells and guard cells. The decrease in cell water potential induces the synthesis and accumulation of abscisic acid (ABA), which triggers stomatal closure, impairs CO_2_ diffusion, causes a sharp drop in the intercellular CO_2_ concentration of mesophyll cells, and may even result in complete stagnation of gas exchange. ATP and NADPH generated on the chloroplast thylakoid membrane during the light reaction stage cannot be effectively consumed due to the lack of sufficient CO_2_ substrates for the dark reactions (Calvin cycle), resulting in massive accumulation of high-energy electrons in the photosynthetic electron transport chain and the induction of redox imbalance, which ultimately triggers chloroplast responses [7,8,9].

Chloroplasts can achieve adaptation through pathways such as osmoprotectant synthesis and the xanthophyll cycle; meanwhile, reactive oxygen species (ROS) can also act as secondary messengers to activate stress defense responses. As ROS continues to accumulate, drought disrupts the balance between ROS production and scavenging in plants, leading to excessive ROS accumulation and subsequent membrane lipid peroxidation. In this process, the ultrastructure of chloroplasts and the orderliness of the membrane system are damaged, the content of photosynthetic pigments decreases significantly, and the functions of photosystem I and photosystem II are impaired [10]. Dynamic fluctuations in calcium concentration, activation of chloroplast retrograde signaling, and interactions with hormonal pathways—such as abscisic acid (ABA)—form the core framework through which chloroplasts perceive and respond to drought stress [11,12]. With the rapid advancement of molecular biology and multi-omics technologies, the self-protection and repair mechanisms of chloroplasts under drought stress have been increasingly elucidated. Antioxidant enzymes—including superoxide dismutase (SOD), ascorbate peroxidase (APX), and catalase (CAT)—along with non-enzymatic antioxidants such as glutathione and ascorbic acid, function synergistically to mitigate oxidative stress [12]. Specific protective proteins, including FBNs and WHIRLY family proteins, help maintain the integrity of the chloroplast membrane system, while the autophagic machinery facilitates the removal of damaged chloroplasts and sustains cellular homeostasis [13]. Moreover, regulators such as melatonin and 5-aminolevulinic acid, as well as the application of nanomaterials, have emerged as promising strategies to enhance drought tolerance in plants; however, their long-term effects and underlying molecular mechanisms remain to be fully elucidated [14].

Plant adaptation to drought stress is a highly coordinated and systemic process involving multiple organs and interconnected signaling pathways. From the initial perception of drought cues to the subsequent adaptive responses within chloroplasts, plants undergo a series of orderly and finely regulated physiological cascade reactions.

## 2. Effects of Drought on Chloroplast Structure and Function

The drought responses of chloroplasts can be broadly categorized into direct and indirect effects (Table 1). Direct effects arise from water deficit–induced osmotic stress that immediately disrupts membrane integrity and chloroplast components. In contrast, indirect effects result from upstream regulatory processes, including stomatal closure, CO_2_ limitation, metabolic feedback, and lipid- or hormone-mediated signaling pathways [15,16,17,18,19].

### 2.1. Changes in Chloroplast Ultrastructure

Chloroplasts, as the primary sites of photosynthesis, possess a highly organized internal architecture comprising thylakoid membranes, grana stacks, and the stroma. Under mild drought stress, chloroplasts activate multiple self-protective strategies to maintain osmotic balance and preserve photosynthetic functionality. Osmotic adjustment—achieved through the synthesis of compatible solutes such as proline, glycine betaine, and soluble sugars—helps equilibrate the osmotic potential between the chloroplast and the cytoplasm. In parallel, ferredoxin plays a key regulatory role in stabilizing electron flow and maintaining redox homeostasis under limited water availability. The xanthophyll cycle also becomes highly important during mild drought, as it dissipates excess excitation energy as heat, thereby preventing photooxidative damage to the photosystems. Meanwhile, the continuous accumulation of high-energy electrons in electron transport chain acceptors begins to affect the thylakoid membrane potential [1]. When the cellular water potential continuously decreases to below −2.0 MPa (a threshold for severe osmotic stress), drought-induced cellular dehydration directly results in chloroplast damage and remodeling [20,21].

Under drought stress, electron microscopy observations have revealed that drought can cause chloroplast shrinkage, contraction or even rupture of the outer membrane, disorganization of grana lamellae, and fragmentation or swelling of thylakoid membranes, ultimately compromising the integrity of the photosynthetic membrane system [22,23]. Meanwhile, the stroma exhibits reduced starch granules, increased membrane permeability, and accumulation of small lytic vesicles, all of which indicate severe subcellular instability of chloroplasts under drought stress.

Alongside damage to photosynthetic membranes, the overall morphology of chloroplasts undergoes pronounced alterations under drought and other stress conditions. Numerous studies have reported structural remodeling of chloroplasts, including transformation from a regular ellipsoidal shape to an irregular form, shrinkage or rupture of membrane systems, folding of thylakoid membranes, and disorganization of grana stacks [24,25].

In addition, several studies have reported ultrastructural variations among different plant species or cultivars under drought conditions. For example, drought-tolerant varieties often maintain partial stability of thylakoid stacking, whereas drought-sensitive varieties exhibit rapid disintegration [26,27,28].

To provide a clearer comparison of drought-induced alterations in chloroplast structure, the major ultrastructural differences under normal and drought conditions are summarized in Table 2.

**Figure 1:**
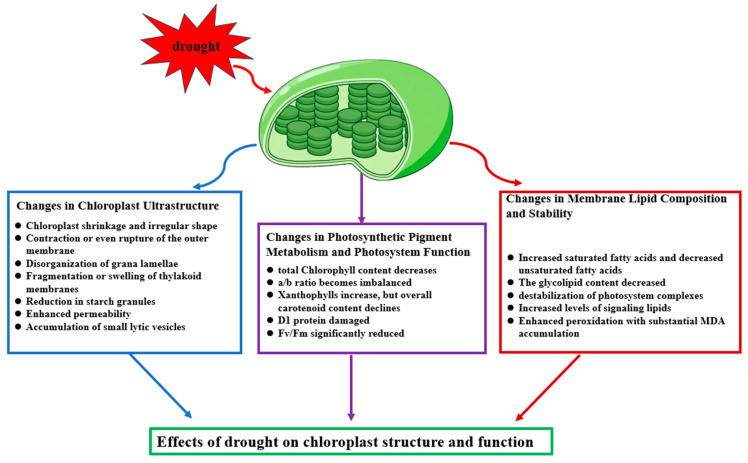
Effects of drought on chloroplast structure and function.

In summary, the disruption of chloroplast ultrastructure represents a critical step in the impairment of the photosynthetic system under drought stress and serves as an important indicator for evaluating drought tolerance among plant species (Figure 1). These structural alterations not only weaken the efficiency of light capture and energy conversion but also provide the initial stimuli for activating downstream signaling and protective mechanisms.

### 2.2. Changes in Photosynthetic Pigment Metabolism and Photosystem Function

Photosynthetic pigments are essential components that enable chloroplasts to capture and convert light energy, and their stability and metabolic balance are critical for the proper functioning of the photosynthetic apparatus. Under drought conditions, both the content and ratio of chlorophylls and carotenoids undergo significant alterations, which are often accompanied by reduced photosynthetic efficiency and aggravated photoinhibition. Studies have shown that drought accelerates the degradation of chlorophyll a and b, leading to a marked decrease in total chlorophyll content and consequently weakening the function of light-harvesting complexes [29]. Meanwhile, carotenoids exhibit a dual role in drought responses: on the one hand, the accumulation of certain carotenoids, such as xanthophylls, enhances energy dissipation and photoprotection; on the other hand, prolonged drought stress results in an overall decline of carotenoids, diminishing the chloroplast’s capacity to scavenge reactive oxygen species (ROS) [30,31]. Under drought conditions, reduced transpiration lowers leaf water potential and impairs chlorophyll biosynthesis. Under combined drought and heat stress, photosystem II (PSII) becomes the primary target [32,33], as the reaction center D1 protein is highly susceptible to oxidative damage, leading to disrupted electron transport and reduced photosynthetic efficiency. In the research on chloroplast chlorophyll fluorescence kinetics, the OJIP technique, leveraging its core advantage of quantifying more than 10 photosynthesis-related parameters, enables more accurate and early diagnosis of the damage inflicted by drought stress on the photosynthetic system, as well as precise localization of the damage sites [34]. In addition, drought also alters the redox state of photosystem I (PSI); under severe water deficit, the reduction levels of electron acceptors on the PSI receptor side—such as ferredoxin (Fd) and NADP^+^—are markedly decreased, resulting in blockage of the electron transport chain and aggravation of the energy imbalance within the chloroplast [10,35].

To alleviate excess excitation energy resulting from pigment loss and impaired electron transport, plants dissipate surplus light energy by regulating non-photochemical quenching (NPQ). Studies have shown that drought stress enhances the activity of the xanthophyll cycle and generally increases NPQ values, thereby reducing photodamage caused by overexcitation of the photosystems [36]. However, excessive energy dissipation can in turn lower the efficiency of light energy utilization, leading to decreased photosynthetic carbon assimilation [37]. Thus, changes in pigment metabolism and photosystem function represent an adaptive trade-off that enables plants to survive under drought conditions, although prolonged or intense stress eventually results in irreversible damage to the photosynthetic apparatus.

It is noteworthy that distinct plant species and genotypes exhibit considerable variation in pigment metabolism and photosystem responses. Drought-tolerant genotypes tend to maintain higher chlorophyll content and stronger carotenoid accumulation during the early stages of drought, together with a faster repair rate of the PSII D1 protein, which collectively contribute to the sustained stability of photosystems. In contrast, drought-sensitive varieties show a rapid decline in pigment levels and severe impairment of photosystem function; once stress intensity exceeds a critical threshold, PSII damage becomes irreversible, leading to growth stagnation and an increased risk of plant mortality [38,39,40,41].

To provide a clearer overview of the combined effects of drought on photosynthetic pigments and photosystem function, the major differences between normal and drought conditions are summarized in Table 3.

In summary, drought stress disrupts chlorophyll and carotenoid metabolism, impairs the functions of both photosystem II and photosystem I, and consequently decreases light energy conversion efficiency while increasing oxidative pressure (Figure 1). The alterations in pigment metabolism and photosystem function are not only direct manifestations of drought-induced damage but also represent adaptive strategies by which plants cope with environmental stress. Understanding these processes helps elucidate the vulnerable components of the photosynthetic system and provides a theoretical basis for identifying drought-tolerant cultivars and developing plant improvement strategies.

### 2.3. Changes in Membrane Lipid Composition and Stability

The structural integrity and functional stability of the photosynthetic membrane system are highly dependent on the composition and fluidity of its lipid matrix. Under drought stress, chloroplasts usually undergo significant adjustments in their membrane lipid composition due to a decrease in water potential [42]. Among the various effects of drought stress, one of the most characteristic features is the alteration in the ratio between phospholipids and glycolipids. Studies have shown that under drought conditions, the content of monounsaturated fatty acids decreases while the proportion of saturated fatty acids increases, thereby reducing membrane fluidity [43,44]. Meanwhile, diacylglycerol-based glycolipids—monogalactosyldiacylglycerol (MGDG) and digalactosyldiacylglycerol (DGDG)—serve as the principal structural lipids of thylakoid membranes. Under drought stress, MGDG levels decline markedly while DGDG content changes relatively little, leading to an increased DGDG/MGDG ratio. This shift is believed to compromise the stability of the thylakoid membrane and weaken the association of photosystem complexes, particularly PSII and PSI [45,46]. Under oxidative stress conditions, membrane lipid peroxidation represents another critical factor affecting chloroplast stability. During drought, excessive accumulation of reactive oxygen species (ROS) triggers lipid peroxidation, leading to elevated levels of metabolites such as malondialdehyde (MDA). These compounds not only serve as markers of membrane lipid damage but also further disrupt chloroplast osmotic balance and membrane potential [47,48].

Conversely, some plant species can mitigate membrane injury by increasing the proportion of unsaturated fatty acids, which slows the rate of lipid peroxidation and enhances membrane stability to a certain extent. Generally, drought-tolerant genotypes maintain higher levels of unsaturated fatty acids and glycolipids during water deficit, thereby preserving thylakoid structure and stabilizing the electron transport chain. In contrast, drought-sensitive cultivars exhibit rapid lipid degradation and enhanced peroxidation, ultimately leading to the collapse of chloroplast membrane systems. These contrasting patterns not only provide molecular markers for identifying drought-resilient genes but also highlight the potential of metabolic engineering targeting membrane lipid pathways to improve drought tolerance in crops [45,49,50,51].

Furthermore, membrane lipid remodeling is closely associated with the dynamic reorganization of the photosynthetic membrane. The drought-induced reprogramming of lipid metabolism not only influences membrane stability but also modulates signal transduction pathways [52]. For instance, certain lipid degradation products—such as phosphatidic acid—function as signaling messengers under stress conditions, participating in the regulation of nuclear gene expression and stress responses [53]. Thus, changes in membrane lipids represent both a manifestation of damage and an integral component of cellular signaling.

To provide a clearer summary of the effects of drought on chloroplast membrane lipid composition and stability, the major alterations under normal and drought conditions are presented in Table 4.

In summary, the imbalance of membrane lipid metabolism and the accumulation of peroxidation products constitute critical pathways of chloroplast damage under drought stress. The remodeling of lipid components not only determines the physical stability of the photosynthetic membrane but also modulates signal transduction and the coordination of stress responses (Figure 1). Future efforts focusing on elucidating the regulatory networks and key factors governing membrane lipid metabolism may provide new molecular targets for enhancing crop drought tolerance.

## 3. Chloroplast Signaling Under Drought Stress

### 3.1. ROS Signaling and Antioxidant Systems

Under drought conditions, the chloroplast is one of the primary sites for reactive oxygen species (ROS) generation. Studies have shown that reactive oxygen species (ROS) are generated through multiple pathways under drought stress. When drought occurs, plant roots first perceive the water deficit and transmit this information to the leaves through the xylem sap. Among the transmitted signals, abscisic acid (ABA) is one of the major root-to-shoot stress signals. Drought induces stomatal closure, decreased photosynthetic rate, imbalanced light energy utilization, and altered chloroplast photochemistry, which in turn leads to excessive ROS production. Within chloroplasts, photosystem I (PSI) and photosystem II (PSII) are the major sites for ROS generation, and photorespiration can also produce ROS [54]. When the photosynthetic electron transport chain is disrupted, excess excitation energy cannot be efficiently utilized, leading to the one-electron reduction of molecular oxygen and the production of various ROS, including superoxide anion (O_2_^−^), hydrogen peroxide (H_2_O_2_), hydroxyl radical (·OH), and singlet oxygen (^1^O_2_) [55,56]. These molecules can cause lipid peroxidation, protein oxidation, and DNA damage, but they also act as signaling messengers that mediate intracellular and intercellular stress responses. In other words, ROS exhibit a dual nature in chloroplast drought responses, functioning both as damaging agents and as critical signal transducers [11,57]. Different types of ROS exhibit distinct mechanisms of action and signaling functions.

The redox homeostasis of chloroplasts relies on a synergistically interacting antioxidant defense network. Ascorbic acid (AsA) and glutathione (GSH), as major water-soluble antioxidants, can accumulate to millimolar concentrations in chloroplasts; they are capable of scavenging reactive oxygen species (ROS) independently and can also metabolize H_2_O_2_ and dissipate excess excitation energy via the AsA–GSH cycle and the water–water cycle. Meanwhile, proteins such as peroxiredoxins (PRX) and glutathione peroxidases (GPX) cooperate with thioredoxin (TRX)-like proteins to participate in ROS scavenging. Among liposoluble antioxidants, carotenoids and tocopherols (Toc) represent the main groups. Toc can accumulate at high levels, scavenge singlet oxygen and lipid peroxyl radicals to inhibit lipid peroxidation, and its oxidized free radicals can be regenerated through AsA and the AsA–GSH cycle. All antioxidant pathways exhibit high interconnectivity and multi-node reciprocal regulation, collectively maintaining the redox homeostasis of chloroplasts [58,59].

Significant differences exist among plant species and genotypes in their capacity to regulate ROS. Drought-tolerant cultivars are able to activate antioxidant enzymes more rapidly and maintain lower levels of malondialdehyde (MDA) accumulation, thereby effectively mitigating damage to chloroplast membranes and photosynthetic proteins. In contrast, drought-sensitive genotypes often experience sustained oxidative stress due to insufficient ROS scavenging [60,61].

Collectively, research on ROS metabolism and antioxidant systems not only reveals the intrinsic connections between chloroplast damage and signaling under drought stress but also provides potential molecular targets for breeding drought-tolerant crops.

### 3.2. Calcium Signaling and Membrane Receptors

Calcium ions (Ca^2+^) play a central role in chloroplast signaling under drought stress. Drought-induced decreases in osmotic potential and the inhibition of photosynthetic electron transport are often accompanied by a rapid rise in Ca^2+^ concentration within the chloroplast stroma and thylakoid lumen. These transient fluctuations in Ca^2+^ levels not only reflect the chloroplast’s perception of environmental stress but also serve as crucial signals that initiate downstream adaptive responses [62,63].

The calcium-sensing receptor (CAS) protein is localized on the outer surface of the thylakoid membrane, where it functions as an essential component of the chloroplast Ca^2+^ signaling machinery. Under drought-induced osmotic stress, CAS contributes to the generation and modulation of stromal Ca^2+^ fluctuations, thereby playing a key regulatory role in chloroplast-mediated drought responses. Previous studies have demonstrated that inactivation of CAS markedly reduces the generation of Ca^2+^ signals, thereby suppressing nuclear gene responses to drought stress [62,64].

At the level of nuclear gene regulation, Ca^2+^-dependent protein kinases and transcription factors play pivotal roles. For instance, Ca^2+^-dependent channels and transporters located on the chloroplast membrane are considered the direct basis for drought-induced Ca^2+^ oscillations. By controlling Ca^2+^ fluxes across membranes, these transport components further amplify or sustain the signaling effects [63,65]. Distinct differences are observed among crop species and genotypes in their Ca^2+^ signaling responses. Drought-tolerant varieties generally exhibit stronger Ca^2+^ oscillation amplitudes and faster signal recovery, enabling the rapid activation of downstream defense responses. In contrast, drought-sensitive varieties often display weaker or delayed Ca^2+^ fluctuations with lower signaling efficiency, ultimately resulting in incomplete chloroplast protection mechanisms [66,67,68]. In summary, Ca^2+^ signaling and membrane receptors serve not only as central components of information transmission during drought adaptation but also as critical determinants of drought tolerance in chloroplasts.

### 3.3. Chloroplast Retrograde Signaling

Chloroplasts function not only as central sites of photosynthesis and metabolism but also as crucial platforms for stress perception and signal transduction. Under drought conditions, chloroplasts can transmit their physiological status to the nucleus through multiple retrograde signaling pathways, thereby regulating the expression of nuclear genes and coordinating whole-cell defense responses. This process, known as retrograde signaling, plays a pivotal role in plant adaptation to drought stress [55].

#### 3.3.1. The GUN Pathway

Among various retrograde signaling pathways, the genomes uncoupled (GUN) pathway is one of the most extensively studied mechanisms. GUN proteins perceive disruptions in the chloroplast photosynthetic electron transport chain and metabolic imbalances, thereby modulating the expression of photosynthesis-associated nuclear genes (PhANGs). Under drought conditions, damage to the photosynthetic membrane system leads to the interruption of electron flow, which subsequently activates the GUN pathway and represses the transcription of photosynthesis-related genes to reduce energy expenditure [69,70]. Among these proteins, GUN1 serves as the central regulatory hub, integrating signals from multiple metabolic processes, including tetrapyrrole biosynthesis, ROS accumulation, and lipid metabolism, to coordinate nuclear gene expression in response to chloroplast stress [71].

#### 3.3.2. The PAP Signaling Pathway

3′-Phosphoadenosine-5′-phosphate (PAP) represents another typical retrograde signaling molecule derived from chloroplasts [72]. Studies have shown that drought stress suppresses phosphatase activity, leading to the intracellular accumulation of PAP. This molecule can traverse the cytoplasm and enter the nucleus, where it regulates gene expression by interfering with the degradation of mRNA between the nucleus and cytoplasm. Through this mechanism, chloroplasts are able to transmit their metabolic stress to the nuclear genome, thereby inducing the transcriptional activation of drought-responsive genes [73]. The PAP signaling pathway is unique in that it is closely associated with post-transcriptional regulation, providing an additional dimension to the chloroplast–nucleus communication network.

#### 3.3.3. The MEcPP Signaling Pathway

The methylerythritol phosphate pathway intermediate, 2-C-methyl-D-erythritol 2,4-cyclodiphosphate (MEcPP), has recently been identified as an important retrograde signaling metabolite. Under drought conditions, MEcPP accumulates significantly within chloroplasts and regulates the expression of defense-related genes by modulating stress-responsive transcription factors in the nucleus [74]. Several studies have revealed that MecPP may interact with hormonal networks, including salicylic acid (SA), jasmonic acid (JA), and abscisic acid (ABA), thereby influencing plant stress response phenotypes [75,76].

#### 3.3.4. Signal Integration and Crosstalk

During drought stress, the chloroplast does not perceive and respond to environmental changes in isolation; rather, it integrates with other cellular pathways through a complex signaling network. Reactive oxygen species (ROS), calcium ions (Ca^2+^), retrograde signals, and plant hormones—particularly abscisic acid (ABA)—collectively form a dynamic and interactive regulatory system [55,77]. The defining feature of this system lies in the coupling and feedback among different signaling components, enabling plants to achieve optimal adaptive strategies under constrained temporal and resource conditions.

ROS serve as one of the central driving forces in the drought-induced signaling network. When photosynthetic electron transport is impeded, chloroplasts generate excessive ROS, which not only act as direct indicators of oxidative stress but also interact with Ca^2+^ dynamics to amplify signaling responses. Studies have revealed that transient ROS elevation can trigger the opening of Ca^2+^ channels, leading to rapid and localized Ca^2+^ oscillations; in turn, these Ca^2+^ fluctuations further activate the antioxidant defense system, forming a bidirectional regulatory loop [78]. This coupling mechanism ensures that ROS does not accumulate uncontrollably but is rapidly perceived and converted into downstream signaling cues.

Ca^2+^ functions as a connector in the interplay of signaling pathways. Numerous studies have demonstrated that Ca^2+^ in the chloroplast signaling network acts not only as a classical secondary messenger mediating short-term stress responses but also as an integrative regulator in retrograde signaling pathways. Under drought conditions, transient elevations in chloroplast Ca^2+^ levels are mediated by the calcium-sensing receptor protein CAS, whose signals can be transmitted to the cytosol and activate MAPK cascades, ultimately targeting the retrograde downstream transcription factor ABI4 to regulate the expression of nuclear genes related to photosynthesis and stress adaptation [79]. This process illustrates that Ca^2+^ signaling can influence the efficiency of chloroplast-to-nucleus communication through inter-compartmental signaling. Meanwhile, GUN1 is recognized as the central integrator of retrograde signaling, capable of sensing multiple upstream inputs derived from photosynthetic electron transport, ROS accumulation, tetrapyrrole metabolism, and membrane lipid remodeling [80].

**Figure 2:**
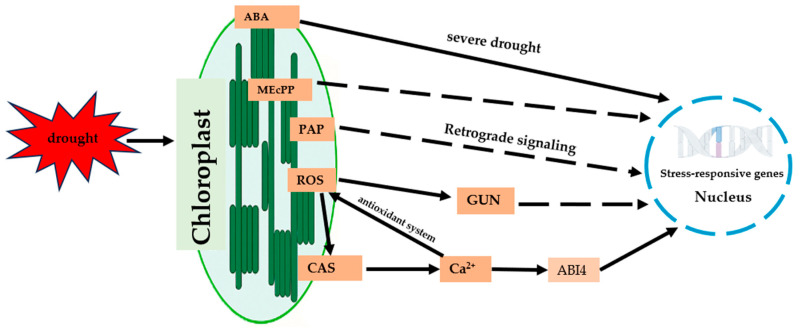
Chloroplast signaling pathways under drought stress.

Retrograde signaling provides an integrative platform within the entire drought-responsive network. Pathways such as the GUN cascade and signaling molecules like PAP and MEcPP convey the chloroplast’s metabolic status to the nucleus, where these signals, together with ROS and Ca^2+^, act on downstream transcription factor networks to regulate the expression of genes associated with antioxidant defense, membrane stabilization, and hormone signaling [11]. Particularly under drought conditions, retrograde signals not only transmit information about chloroplast damage but also engage in extensive crosstalk with the abscisic acid (ABA) signaling pathway. Previous studies have demonstrated that the rate of ABA biosynthesis is frequently regulated by the chloroplast’s functional state, while ABA signaling, in turn, interacts with retrograde pathways by modulating gene expression, thereby forming a positive feedback mechanism that reinforces stress adaptation [73,77]. This multi-signal interactive system reveals the hierarchical and complex nature of drought-stress responses. Compared with single signaling pathways, network interactions not only enhance the redundancy and reliability of signal transmission but also allow plants to flexibly adjust their response strategies according to the intensity of stress. For instance, transient coupling between ROS and Ca^2+^ under mild drought is sufficient to activate localized defense responses, whereas severe drought requires the sustained participation of retrograde and ABA signaling to mobilize systemic resources for survival. Such flexibility represents an adaptive mechanism that has been refined through long-term plant evolution [72,81]. This multilayered system enables plants to fine-tune stress perception, response intensity, and resource allocation through reciprocal feedback among distinct signaling modules (Figure 2).

## 4. Chloroplast Protection and Repair Mechanisms

### 4.1. Protein Protection and Regulation

Under drought stress, chloroplast proteins are highly vulnerable to structural damage and functional loss. Thylakoid membrane proteins and stromal soluble proteins are particularly sensitive, including the core subunits of the photosynthetic complexes and key enzymes involved in carbon assimilation. To maintain chloroplast homeostasis, plants have evolved a sophisticated system of protein protection and regulatory mechanisms that mitigate damage and promote repair [10]. The FBN protein family constitutes a group of structural proteins localized on the thylakoid membranes and plastoglobules within chloroplasts. Studies have shown that FBNs stabilize pigment–protein complexes and enhance photoprotective capacity by modulating carotenoid binding. Under drought stress, the accumulation of FBNs helps to alleviate photoinhibition and excessive ROS generation, thereby improving the tolerance and stability of the photosynthetic system [82,83].

Another key group of regulatory factors is the WHIRLY protein family, which consists of single-stranded DNA-binding proteins capable of shuttling between the chloroplast and the nucleus. Within chloroplasts, WHIRLY proteins primarily function to maintain genome stability and mitigate DNA oxidative damage. During drought stress, they exhibit dual functions by enhancing ROS scavenging and activating the expression of defense-related genes [13,84].

Similarly, pentatricopeptide repeat proteins (PPRs) play essential roles in RNA editing and stabilization within chloroplasts. Drought-induced abnormalities in RNA editing often lead to reduced translational efficiency of photosynthetic enzymes, whereas the regulatory activity of PPRs can partially restore this process, thereby maintaining chloroplast functionality [85,86]. In maintaining protein homeostasis, the cooperation between molecular chaperones and proteolytic systems is equally crucial. Molecular chaperones such as HSP70 and HSP90 rapidly bind to damaged proteins under drought conditions, preventing their aggregation and assisting in refolding and repair [87]. Meanwhile, chloroplast-localized serine proteases and metalloproteases are responsible for degrading irreversibly damaged proteins, thereby creating space for the synthesis and assembly of newly formed polypeptides [88,89]. This hierarchical regulation mode, comprising protection, repair, and degradation, enables chloroplasts to maintain a minimal yet essential level of functionality even under extreme environmental stress.

The chloroplast protein protection and regulatory system serves as an indispensable defensive barrier during plant adaptation to drought. Through the coordinated functions of specific proteins such as FBNs, WHIRLYs, and PPRs, together with the synergistic actions of molecular chaperones and proteases, plants are able to maintain chloroplast homeostasis and sustain photosynthetic performance under stress conditions. Elucidating these regulatory components and their interactions will provide valuable insights for the molecular improvement of drought-tolerant crops.

### 4.2. Antioxidant Systems and ROS-Scavenging Mechanisms

Under drought stress, the excessive accumulation of reactive oxygen species (ROS) within chloroplasts is a primary cause of cellular oxidative stress and structural damage. To maintain redox homeostasis, plants have evolved a comprehensive antioxidant system comprising both enzymatic and non-enzymatic components. The coordinated action of these two branches not only determines the efficiency of ROS detoxification but also influences the sensitivity and stability of redox-related signaling pathways [12,90,91].

The enzymatic antioxidant system constitutes the core component of ROS detoxification. GR regenerates reduced GSH, thereby sustaining the efficient operation of the ascorbate–glutathione (ASA–GSH) cycle [92,93]. This cycle is considered one of the most important ROS-scavenging mechanisms within chloroplasts, maintaining high detoxification efficiency even under prolonged drought stress.

Complementing the enzymatic reactions, non-enzymatic antioxidants play equally vital roles in regulating redox balance. Molecules such as ASA, GSH, tocopherols (vitamin E), carotenoids, and flavonoids are crucial in scavenging singlet oxygen and hydroxyl radicals.

Distinct species and genotypes exhibit substantial differences in their capacity to regulate antioxidant systems. Drought-tolerant plants typically respond rapidly during the early stages of stress by enhancing the activities of key enzymes such as SOD and APX, while maintaining elevated levels of ASA and GSH. This enables them to alleviate oxidative pressure effectively before structural damage occurs. In contrast, drought-sensitive varieties often display delayed antioxidant responses, leading to intensified chloroplast membrane lipid peroxidation and a marked increase in malondialdehyde (MDA) accumulation. Together with the preceding discussion on ROS signaling, these findings highlight that the antioxidant system functions not only as the primary executor of ROS detoxification but also as a fundamental prerequisite for the precise regulation of ROS-mediated signaling [94,95]. The major members and their functional roles are summarized in Table 5.

### 4.3. Functional Regulators

Recent studies have demonstrated that various small signaling molecules, phytohormone analogs, and novel nanomaterials can enhance drought tolerance by modulating chloroplast structure and function. These substances help stabilize the photosynthetic apparatus and improve ROS-scavenging capacity, thereby strengthening overall photosynthetic performance and stress resilience in plants [96].

Melatonin, an indolamine molecule present in plants, has been shown to effectively enhance plant resistance to adverse environmental conditions, including drought stress. As one of the main sites for endogenous melatonin biosynthesis in plants, chloroplasts exhibit a significant increase in the activity of melatonin synthases under drought–heat combined stress, driving the rapid accumulation of endogenous melatonin and providing a core material guarantee for chloroplasts to adapt to stress. In terms of antioxidant defense, endogenous melatonin can directly scavenge hydroxyl radicals (·OH) and superoxide anions (O_2_^−^) generated in chloroplasts, while effectively inducing the activity of antioxidant enzymes such as superoxide dismutase (SOD) and ascorbate peroxidase (APX) in chloroplasts. This further prevents lipid peroxidation of the thylakoid membrane and maintains the redox balance within chloroplasts. Regarding structural protection, endogenous melatonin enhances the stability of the thylakoid membrane, reduces the increase in membrane permeability, and maintains the structural stability of photosynthetic pigments. Meanwhile, it effectively alleviates stress-induced damage to photosystems (especially Photosystem II, PSII) and improves the overall structural integrity of chloroplasts by regulating the reversible phosphorylation of thylakoid proteins and protecting core components of PSII, such as the D1 protein. At the level of metabolic and hormonal crosstalk, melatonin can form an antagonistic interaction with abscisic acid (ABA). It avoids CO_2_ deficiency caused by excessive stomatal closure by downregulating the expression of key factors in the ABA signaling pathway and ABA biosynthesis-related genes, thereby providing sufficient substrates for the Calvin cycle (dark reactions) in chloroplasts. This balances the plant’s water conservation needs with the photosynthetic function of chloroplasts, prevents energy excess and excessive reactive oxygen species (ROS) accumulation induced by the imbalance between light reactions and dark reactions, and comprehensively supports the structural integrity and functional stability of chloroplasts under drought–heat combined stress.

5-Aminolevulinic acid (ALA), a key precursor in chlorophyll biosynthesis, plays an important role in enhancing chloroplast performance under drought stress. Exogenous application of ALA promotes chlorophyll pigment synthesis and accelerates the repair of the damaged D1 protein, thereby improving the tolerance of photosystem II (PSII). In drought-stress experiments, ALA-treated plants exhibit a slower decline in chlorophyll content and a prolonged maintenance of net photosynthetic rate, indicating its unique protective role in sustaining chloroplast stability and photosynthetic efficiency [97,98].

Hydrogen sulfide (H_2_S), a gaseous signaling molecule, also plays a crucial role in plant responses to drought stress. Exogenous application of H_2_S donors significantly increases the degree of unsaturation in chloroplast membrane lipids, reduces lipid peroxidation levels, and alleviates the accumulation of malondialdehyde (MDA), thereby contributing to improved membrane stability under stress conditions [99]. In addition, H_2_S interacts with the abscisic acid (ABA) signaling pathway to facilitate stomatal regulation and enhance water-use efficiency, thereby indirectly alleviating drought-induced damage to chloroplasts [100].

In recent years, the application of nanomaterials has opened new avenues for chloroplast protection. Studies have shown that nanoparticles such as zinc oxide (ZnO) and titanium dioxide (TiO_2_) can enhance chloroplast light-harvesting efficiency and electron transport capacity at low concentrations. The application of ZnO nanoparticles in crops such as rice and maize has been reported to improve pigment stability, promote thylakoid membrane stacking, and activate the antioxidant system [101,102,103]. However, the potential toxicity and environmental risks of nanomaterials still require systematic evaluation, and their practical applicability remains to be further explored. To provide a clear overview of the effects of endogenous and exogenous compounds, the major categories and their primary functions are summarized in Table 6.

Overall, regulatory compounds markedly enhance the ability of chloroplasts to maintain homeostasis under drought stress through multilayered and multipathway mechanisms. These compounds not only directly strengthen the antioxidant system and stabilize the photosynthetic apparatus, but also interact with hormonal signaling and lipid metabolism pathways, providing new perspectives for drought-resistance research [96,104]. However, the molecular targets, long-term effects, and field applicability of different compounds remain insufficiently understood. Future studies integrating molecular biology and field experimentation are required to further validate their potential in agricultural applications.

### 4.4. Chloroplast Autophagy and Degradation

Under prolonged drought stress, parts of the chloroplast structure and function may experience irreversible damage. In such cases, plants selectively remove inactivated or damaged chloroplasts through autophagic and degradative pathways to maintain overall cellular homeostasis [105]. Autophagy, a conserved intracellular degradation mechanism, manifests at the chloroplast level mainly as chlorophagy and the formation of intraplastidic autophagic bodies. Electron microscopy observations have revealed that under drought conditions, chloroplasts or their fragments are frequently enclosed by double-membrane vesicles. These autophagic bodies are subsequently transported to the vacuole for degradation, thereby preventing damaged chloroplasts from becoming continuous sources of reactive oxygen species (ROS) [105].

Chloroplast autophagy is not merely a passive clearance process but is accompanied by a series of finely tuned regulatory events. Studies have demonstrated that the autophagy-related gene (ATG) family plays a central role in this process, with its encoded proteins participating in the formation and transport of autophagosomes. Under drought-inducing conditions, the number of autophagosomes labeled with ATG8 proteins increases markedly, indicating that the autophagic machinery remains highly active during chloroplast turnover [106]. On the other hand, certain degradation processes are not dependent on canonical autophagy but are instead mediated through plastid–internal degradation pathways [107]. For instance, residual proteins in the chloroplast stroma can be degraded by plastid-localized serine proteases, a process known as the plastidial degradation pathway. This mechanism plays a vital role in regulating chloroplast longevity and maintaining proteostasis during drought stress [108].

Moreover, autophagy is closely interconnected with multiple signaling molecules. Drought-induced accumulation of ROS is often regarded as a key signal that triggers the initiation of autophagy, while fluctuations in Ca^2+^ concentration and the abscisic acid (ABA) signaling pathway can modulate autophagy levels by regulating ATG protein activity [64]. This evidence suggests that autophagy is not merely a degradative mechanism but an integral component of the chloroplast–nucleus–hormone signaling network. Through the selective degradation of severely damaged chloroplasts, plants can recycle cellular resources and ensure the efficient operation of the remaining chloroplasts under drought conditions [109,110]. Differences in autophagic and degradative capacities among genotypes are directly associated with their levels of drought tolerance. Drought-tolerant varieties typically exhibit higher autophagic activity, enabling the early removal of irreversibly damaged chloroplasts and thereby reducing overall oxidative stress. In contrast, genotypes with impaired autophagy tend to accumulate large numbers of damaged chloroplasts, leading to sustained ROS elevation and exacerbated cellular injury. Consequently, chloroplast autophagy and degradation represent not only protective mechanisms but also key indicators of drought resilience in plants [111,112].

**Figure 3:**
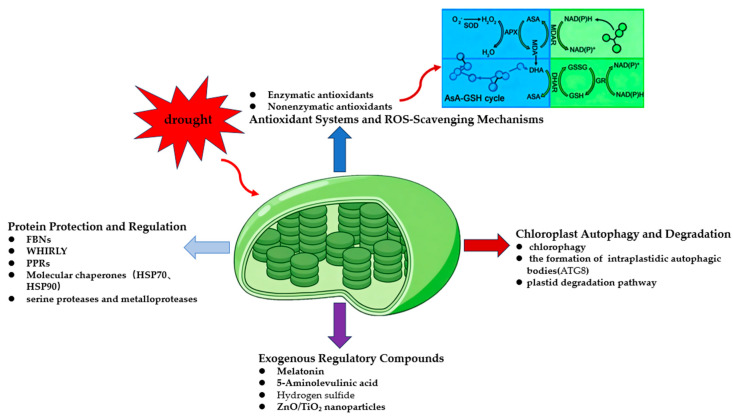
Chloroplast protection strategies and repair mechanisms.

To illustrate the integration of these protective pathways, we present a schematic diagram in Figure 3. As shown in the figure, different protective mechanisms coordinate with one another to provide optimal protection for chloroplasts under drought stress, thereby maintaining cellular homeostasis and enabling plants to achieve the best possible growth through an adaptive trade-off strategy.

## 5. Perspectives and Future Directions

### 5.1. Systematic Analysis of Chloroplast Signaling Networks

Under drought stress, the chloroplast functions not only as a vulnerable target of damage but also as a central hub coordinating whole-plant responses. Current research on ROS, Ca^2+^, retrograde signaling, and hormonal pathways has largely focused on their individual roles, lacking an integrated understanding of their network-level interactions. Future studies should therefore emphasize the systematic dissection of signaling network coupling, particularly focusing on the dynamic temporal and spatial patterns that underlie chloroplast-mediated stress regulation.

In actual stress responses, different signaling molecules rarely act in isolation but rather operate in a pulse-like or stepwise sequence of interlinked events. For example, transient ROS accumulation may serve as a trigger for Ca^2+^ fluctuations, while subsequent Ca^2+^ changes can influence the transmission of retrograde signals to nuclear genes. Such hierarchical signaling implies that a multilayered “dialogue system” exists within the chloroplast, enabling rapid perception and progressive amplification of external stimuli through inter-signal coupling.

From a methodological perspective, traditional molecular approaches alone are insufficient to unravel these intricate relationships. The integration of multi-omics datasets together with advanced dynamic imaging technologies is expected to be pivotal for future breakthroughs. By correlating chloroplast metabolic states, signaling trajectories, and gene expression responses in a unified framework, researchers may be able to construct network models that more closely represent the in vivo signaling landscape.

More importantly, this systems-level understanding should not remain confined to basic research. Identifying key regulatory nodes within these networks and leveraging them in crop improvement could substantially enhance plant drought adaptability. Therefore, a comprehensive understanding of chloroplast signaling networks represents not only a fundamental requirement for elucidating stress mechanisms but also a critical pathway toward molecular breeding and agricultural applications.

### 5.2. Applications of Regulatory Compounds and Novel Materials

In drought-stress research, the application of regulators has increasingly been recognized as an effective strategy for enhancing crop drought tolerance. By directly modulating chloroplast function, endogenous and exogenous regulation can rapidly stabilize the photosynthetic apparatus and alleviate oxidative damage. Small signaling molecules such as melatonin, ALA, and H_2_S have shown positive effects in experiments, often acting by enhancing antioxidant activity, slowing pigment degradation, and stabilizing membrane structures to help plants endure periods of water deficiency. Compared with genetic modification, such approaches are easier to implement and offer greater practical feasibility in agricultural production.

Meanwhile, the emergence of novel nanomaterials has opened new perspectives for chloroplast protection. Low concentrations of nanoparticles such as ZnO or TiO_2_ have been reported to improve light-harvesting efficiency and positively influence membrane lipid stability. The potential of nanomaterials lies not only in enhancing photosynthetic performance but also in serving as carriers for exogenous molecules, allowing controlled and localized release. This may overcome the limitations of conventional treatments, which often exhibit short duration and low efficiency.

However, several challenges remain regarding the application of endogenous and exogenous compounds. Determining optimal dosages for different crops and environments, and assessing the long-term ecological risks of material residues, are critical issues that must be addressed. We propose that combining endogenous and exogenous treatments with molecular breeding strategies could yield complementary benefits—endogenous and exogenous compounds provide immediate protection, while genetic modification ensures long-term stability. Coordinated application of the two approaches may ultimately achieve synergistic improvements in crop drought resistance.

### 5.3. Prospects for Molecular Breeding

As the critical role of chloroplasts in drought response mechanisms becomes increasingly clear, translating these research findings into practical molecular breeding strategies has become particularly important. The multifaceted functions of chloroplasts in photosynthetic energy conversion, signal transduction, and self-protection make them a highly promising target for genetic improvement. Future breeding efforts should move beyond the enhancement of single traits and instead focus on improving overall adaptability through coordinated multigene regulation.

We propose that the core nodes within chloroplast-associated signaling networks may represent the most promising targets for application. Precise manipulation of key genes related to ROS detoxification, Ca^2+^ dynamics, or retrograde signaling could enhance the defensive efficiency of crops under drought conditions. Moreover, the integration of gene-editing technologies to optimize chloroplast functional proteins may overcome the limitations of traditional breeding, such as long development cycles and low efficiency.

Gene-editing technology has established a multi-level and high-precision plant drought-resistance improvement system through the “local functional optimization” of targeted chloroplast genomes and the “global network regulation” of nuclear genomes. In recent years, technological breakthroughs (such as chloroplast-based editing systems, in situ promoter modification, and nucleus–chloroplast dual-targeted editing) have addressed the key bottlenecks of traditional breeding, including resistance–growth imbalance, long breeding cycles, and low efficiency, providing an unprecedented technical tool for crop drought-resistance breeding. In the future, with the development of multi-omics data integration (genomics, transcriptomics, metabolomics) and intelligent editing tools (AI-assisted target design, automated screening platforms), gene-editing technology will achieve a leap from “single-gene editing” to “genome-wide precise design.” This will foster the development of new crop varieties with high drought resistance, high yield, and strong environmental adaptability, providing core technical support for addressing global climate change and ensuring food security.

Overall, chloroplasts play an important role in drought stress responses, and they can serve as a key target for enhancing drought resistance as part of strategies for drought-resilient breeding. With the ongoing advancement of multi-omics technologies and precision genome-editing tools, chloroplast-centered molecular design is poised to lead the next phase of crop improvement and provide a robust foundation for sustainable agriculture.

## Figures and Tables

**Table 1:** Direct vs. indirect effects of drought on chloroplast structure and function.

Category of Effects	Specific Affected Phenomena	Direct/Indirect Effect
Chloroplast Ultrastructure	Reduced volume, membrane rupture, disorganized thylakoids	Direct effect
Stroma alterations	Decrease in starch grains, increase in lytic vesicles	Direct effect
Chlorophyll degradation	Reduced Chl a/b	Indirect effect
Carotenoid changes	Increase in xanthophylls/overall decline	Mainly indirect effect
PSII damage	D1 oxidation, decreased Fv/Fm	Indirect effect
PSI inhibition	Reduced Fd/NADP^+^	Indirect effect
Increased NPQ	Enhanced energy dissipation	Indirect effect
Membrane lipid changes	Decrease in MGDG, increased saturation	Direct effect
Lipid peroxidation	Increased MDA	Indirect/mixed effect

**Table 2:** Typical ultrastructural changes in chloroplasts under drought stress.

Feature	Normal Conditions	Drought Conditions
Chloroplast morphology	Ellipsoidal and structurally intact	Irregular shape with reduced volume
Thylakoid lamellae	Well-organized with distinct grana	Disrupted or swollen; grana disintegration
Outer membrane	Intact double-membrane structure	Constricted or ruptured; increased permeability
Stroma	Rich in starch granules; few osmotic vesicles	Reduced starch granules; accumulation of small vesicles

**Table 3:** Major changes in photosynthetic pigments and photosystem function under drought conditions.

Parameter	Normal Conditions	Drought Conditions
Chlorophyll content	Stable levels with a normal a/b ratio	Total content decreases; a/b ratio becomes imbalanced
Carotenoids	Moderate levels with normal photoprotective activity	Xanthophylls increase, but overall carotenoid content declines
Photosystem II (PSII)	D1 protein remains stable; Fv/Fm at normal level	D1 protein damaged; Fv/Fm significantly reduced
Photosystem I (PSI)	Smooth electron transport; active reduction of Fd and NADP^+^	Electron transport impeded; reduced capacity of Fd and NADP^+^
Energy dissipation (NPQ)	Maintained at basal level	NPQ enhanced, improving photoprotective capacity

**Table 4:** Changes in chloroplast membrane lipid composition and stability under drought stress.

Parameter	Normal Conditions	Drought Conditions
Fatty acid composition	Higher proportion of unsaturated fatty acids	Increased saturated fatty acids; decreased unsaturated fatty acids
Glycolipids (MGDG, DGDG)	Stable content supporting thylakoid stacking	Decreased levels; destabilization of photosystem complexes
Signaling lipids (e.g., phosphatidic acid)	Low baseline levels maintaining homeostasis	Elevated levels involved in stress signaling regulation
Lipid peroxidation	Low peroxidation; normal MDA content	Enhanced peroxidation with substantial MDA accumulation

**Table 5:** Major components and functions of the chloroplast antioxidant system under drought conditions.

Component Type	Major Members	Functional Roles
Enzymatic antioxidant	SOD, APX, CAT, GR	Eliminate O_2_^−^ and H_2_O_2_; maintain the ASA–GSH cycle
Non-enzymatic antioxidants	ASA, GSH, tocopherols, carotenoids, flavonoids	Scavenge ^1^O_2_ and ·OH; dissipate excess excitation energy
Cooperative mechanisms	ASA–GSH cycle, ROS signaling regulation	Balance ROS scavenging and signal transduction
Lipid peroxidation	Low level, normal MDA content	Enhanced peroxidation and substantial MDA accumulation

**Table 6:** Major effects of regulatory compounds on chloroplasts under drought conditions.

Regulators	Primary Effects	Features
Melatonin	Enhances antioxidant enzyme activities and maintains thylakoid membrane integrity	Scavenges ROS and delays photosystem damage
ALA (5-aminolevulinic acid)	Promotes chlorophyll synthesis and accelerates D1 protein repair	Stabilizes PSII and improves photosynthetic rate
H_2_S (Hydrogen sulfide)	Increases membrane lipid unsaturation and reduces MDA accumulation	Mitigates lipid peroxidation and interacts with ABA signaling
ZnO/TiO_2_ nanoparticles	Enhances pigment stability and promotes electron transport	Improves light-harvesting efficiency and activates the antioxidant system

## Data Availability

No new data were created or analyzed in this study. Data sharing is not applicable to this article.
